# The Challenge of Treating Abdominal Aortic Aneurysms with Hostile Neck Anatomy: An Overview

**DOI:** 10.3390/jcm13051460

**Published:** 2024-03-02

**Authors:** Alex Houser, Camilo Martinez, Apostolos Tassiopoulos

**Affiliations:** 1 Division of Vascular Surgery, Department of Surgery, Stony Brook University Hospital, Stony Brook, NY 11794, USA; alex.houser@stonybrookmedicine.edu (A.H.); camilo.martinez@stonybrookmedicine.edu (C.M.); 2 Department of Surgery, Division of Vascular and Endovascular Surgery, Stony Brook Medicine Health Sciences Center, T-19, Room 020, Stony Brook, NY 11794, USA

**Keywords:** abdominal aortic aneurysm, hostile anatomy, hostile neck, endovascular aortic aneurysm repair, endoleak, graft migration

## Abstract

Hostile aortic neck anatomy challenges the outcomes of endovascular abdominal aortic aneurysm repair (EVAR). Besides reverting to open surgical repair (OSR), thoughtful endograft selection and a number of advanced endovascular techniques have been suggested as potential solutions for preventing proximal seal zone complications, improving EVAR durability, and preventing aneurysm-related death. Each technique is associated with advantages and limitations and there has not been a credible direct comparison amongst them in the form of a well-designed prospective trial. The not infrequent presence of multiple hostile anatomic characteristics further complicates decision making and challenges the surgeon’s skills. This paper serves as an overview of hostile neck anatomy and its implications on EVAR. We provide a concise literature review with the purpose of outlining the treatment modalities and outcomes in this patient population.

## 1. Purpose

Our aim in this paper was to review publications from 1995–2023 discussing the impact that hostile aortic neck anatomy has on the outcomes of EVAR, with the goal of providing insight into current management. The overview primarily emphasized two key aspects of management. First, we aimed to provide a concise definition of a hostile aortic neck based on the current literature. A second keypoint was to review the devices used in current practice and adjunctive techniques to address a short aortic neck with particular emphasis on the use of the Heli-FX™ EndoAnchor™ System.

## 2. Literature Review

A literature review was conducted using PubMed using the search terms (“endovascular aortic aneurysm” or “abdominal aortic aneurysm” or “endovascular aneurysm”) and (“endovascular” or “repair”) and (“hostile neck” or “aortic neck” or “neck angulation” or “neck dilation” or “conical neck”). An additional search was performed using the terms “endoanchor” or “endoanchors” or “heli-fx”. Once articles were selected, the reference lists from these articles were reviewed to identify any additional qualifying studies.

The search was limited to 1 January 1996 through November 2023. Only English language articles were included. We selected articles pertaining to abdominal aortic aneurysm repair in the context of challenging neck anatomy. Articles that focused on other challenging anatomic factors including vascular access and iliac aneurysmal disease were excluded.

Our search yielded 801 results. The titles and abstracts were analyzed by two of the authors (AH and CM). Of the articles, 36 were chosen for inclusion in our review. Another 17 were identified through review of the aforementioned reference lists, and one was from a recent podium presentation at a large vascular conference.

## 3. Introduction

### 3.1. What Is a Hostile Neck?

Proximal aortic neck is defined as the length of healthy, parallel wall aorta from the most caudal renal artery to the proximal extent of the abdominal aortic aneurysm (AAA). The distal extent of the aortic neck is defined as the point where the aortic diameter exhibits less than a 10% increase when compared to the diameter at the level of the lowest renal artery. The general definition of “hostile neck” is one whose anatomy makes a standard EVAR repair with a commercially available endograft suboptimal. The term “hostile neck” was first introduced by Dillavou [1] in 2003 and has since been one of the most debated topics in the endovascular repair of AAA. Its definition is not clearly established. The most widely accepted hostile neck characteristics based on the Delphi Consensus Group (Italy, 2017) [2] are listed in Table 1. Some suggest that any aortic neck that does not meet IFU criteria should be considered hostile and, in fact, the anatomy of aneurysms with hostile necks often puts them “outside of instructions for use (IFU)” for some or all of the off-the-shelf endografts on the market. An overview of the IFU for devices available in the USA in 2023 is provided in Table 2. Although the definition is still debated, the significance of hostile neck anatomy is well established. Several studies have shown that standard EVAR in these patients is associated with a higher rate of both immediate and remote proximal seal zone failures manifesting as type Ia endoleaks and a higher rate of aneurysm-related mortality [3,4]. In addition, the presence of more than one hostile characteristic multiplies the risk of immediate or remote EVAR complications [3]. Despite these findings, in routine clinical practice a significant percentage of elective EVARs is performed outside of IFU. A report using the M2S, Inc, imaging database has suggested that in a cohort of 1736 patients from 2000–2010, 58.1% were IFU adherent and 41.9% IFU nonadherent [5]. Aortic neck length and angulation were the two most common culprits of non-compliance, accounting for 62.4% and 10.2% of the IFU-nonadherent group, respectively. Similarly, in a different cohort of 10,228 patients undergoing EVAR, Schanzer et al. demonstrated that only 42% of the patients had anatomy that met the strict definition of device IFU and only 69% met a more liberal definition [6], suggesting that EVAR device IFU compliance is suboptimal.

The need for advanced solutions that would effectively address some of the challenges of hostile necks has led to the development of new custom-made endografts (fenestrated and branched devices) and procedure modifications (parallel grafts, physician-modified grafts) that extend the length of the proximal seal zone, as well as adjuncts (endoanchors) that are designed to improve endograft fixation and seal.

### 3.2. Why Is a Hostile Neck Relevant?

Endograft seal and fixation are the most important parameters in the success and durability of EVAR. Fixation refers to the method by which an endograft maintains its original deployed position, whereas sealing is a graft’s ability to exclude blood flow into the aneurysm sac. Consequently, fixation failure results in graft migration and inadequate sealing generates an endoleak [7], both of which are the primary means of endograft failure as it relates to the proximal neck. An infrarenal AAA neck that is straight, uniform, at least 15 mm long, and free of thrombus or calcification is considered optimal for proximal endograft fixation and sealing. Fixation failure with device migration may precipitate a sealing defect. However, even with adequate fixation, proximal seal failure can still be present. It is important to understand the combined effect of both mechanisms in the context of proximal neck length, angulation, and diameter. EVAR in patients with hostile neck characteristics is associated with higher rates of both early and late type Ia endoleaks [3], suggesting a limited durability of standard endovascular repair in these anatomies. It is also important to emphasize that the more hostile neck characteristics that are present, the higher the risk of EVAR failures [3] and this should weigh heavily in the decision regarding the optimal treatment approach. Finally, approximately 25% of all EVAR patients develop dilatation of the aortic neck diameter and/or the common iliac arteries post-endograft implantation, which further threatens the durability of the endograft seal zones [8,9]. Proximal seal zone failures increase the rate of reinterventions and the risk of AAA rupture after EVAR, contributing to a higher rate of aneurysm-related mortality [3,4]. This evidence underscores the need for continuous follow-up of all EVAR patients, with adequate imaging not only of the aortic sac but of the seal zones as well.

## 4. Hostile Neck Characteristics

### 4.1. A Short Proximal Neck

For most commercially available endografts, a neck length of 15 mm or longer is required on the basis of IFU; however, in clinical practice, 10–15 mm is the generally accepted minimum neck length for achieving infrarenal fixation. Individuals with shorter AAA necks have a reduced contact surface, resulting in less frictional force and radial force along the length of the neck, and are therefore predisposed to graft displacement and endoleaks [10]. Neck lengths shorter than 15 mm are associated with higher rates of early and late type Ia endoleaks and need for secondary intervention [11]. Some of the newer devices have been approved for shorter neck lengths. Specifically, the Endurant^™^ II/Endurant^™^ IIs Stent Graft System (Medtronic, Minneapolis, MN, USA) and the Ovation Alto^®^ Abdominal Stent Graft System (Endologix LLC, Irvine, CA, USA) require minimum neck lengths of 10 mm and 7 mm, respectively [12]. Devices used outside of IFU guidelines have demonstrated a similar incidence of type Ia endoleaks in the short term [13]. In long-term follow-up (3–8 years), however, a short proximal neck remains independently associated with type I endoleaks in standard EVAR [14].

Almost every modern endograft design has included features that help achieve fixation such as bare-metal stents extending to the suprarenal aorta, barbs that engage the aortic wall, or columnar support [15]. The suprarenal aorta is relatively resistant to dilatation after EVAR and usually has less disease compared to the infrarenal segment [16]. Suprarenal bare-metal stenting has not been shown to put patients at risk of worsening kidney dysfunction clinically, nor has this been demonstrated in animal models [17]. Hooks or barbs mechanically embed the graft into the aortic wall in order to mitigate the risk of caudal migration. On the other hand, columnar support provides additional structural stability to the body of the endograft and therefore helps prevent device migration. The Endologix AFX device not only has a metal frame that adds structural body support to the endograft, but is also designed to be based on passive fixation by having the flow divider sit directly on the aortic bifurcation. This comes at the expense of a stiff main body that only partially accommodates aortic angulation. For that reason, in recent years, manufacturers primarily aim to produce grafts with more flexibility.

### 4.2. Neck Angulation

Severe aortic neck angulation (≥60 degrees) is associated with perigraft endoleaks secondary to poor proximal sealing and graft separation [18]. Increasing proximal angulation decreases the pull-down force necessary to keep the graft from dislodging [19]. In first-generation endografts, a correlation between increased neck angulation and an increased incidence of aneurysm expansion, device migration, and type I endoleaks has been reported [20]. The prevailing notion was that larger angles increase the stress on the device and therefore are at risk of migration, but the recent literature has called this into question [21,22]. This metric has been criticized because it attempts to use a two-dimensional value to define a dynamic, three-dimensional feature—a limitation several authors have acknowledged. However, aortic curvature (defined by bending rate and tortuosity throughout the entire sac) is a predictor of intra-operative and late type Ia endoleaks. The underlying mechanism is that increased curvature results in more traction and tilt on the proximal stent graft [23,24]. Nevertheless, the IFU for most current devices recommends an angulation of less than 60 degrees, with only a few exceptions. Two suprarenal platforms are approved for up to 75 degrees: the Treovance^™^ (Terumo Aortic, Sunrise, FL, USA) and the Endurant II^™^ (Medtronic Cardiovascular, Santa Rosa, CA, USA) systems. The infrarenal systems Anaconda^™^ (Terumo Aortic, Glasgow, UK), Aorfix^™^ (Lombard Medical, Didcot, UK), and the Conformable Excluder C3 device^™^ (WL Gore & Associates, Flagstaff, AZ, USA) extend their IFU up to 90 degrees [25]. Midterm data for the Aorfix device show promising results, demonstrating no difference in sac expansion or regression in necks ≥ 60 degrees [26].

### 4.3. Neck Diameter

In order to achieve and maintain an adequate seal, the proximal endograft must have circumferential apposition to a healthy segment of aortic wall. Therefore, the relationship between the endograft size and AAA neck diameter is critical for a durable EVAR. Aortic neck diameter has been defined as the diameter, measured from the outer walls, at the lowest renal artery in an orthogonal plane (i.e., in a plane at a right angle to the centerline of the lumen) [6]. This is the accepted definition when the neck walls are parallel; in a conical-shaped neck, however, the definition of neck diameter is less clear and some surgeons have suggested that the more applicable diameter is the average of the smaller and larger diameters along the proximal neck. For most surgeons, computed tomographic angiography (CTA) is the modality of choice for preoperative evaluation; however, this generates only a static image of the aorta at a random point in the cardiac cycle [27]. Diameter changes in the AAA neck of 0.9–2.4 mm with systole have been reported; thus, one of the primary limitations of CTA is the inability to assess dynamic changes in aortic distention, which could lead to incorrect graft sizing [28]. However, the clinical relevance of dynamic imaging (with duplex ultrasound and/or intravascular ultrasound) is not clear and CTA remains the gold standard for preoperative imaging.

There is no clear definition of a wide proximal neck. Most studies have used 28 mm as a threshold for comparison, but in different reports this threshold ranges between 25 and 31 mm [29,30,31,32]. Infrarenal neck diameters ≥28 mm are associated with an increased risk of type Ia endoleaks, AAA sac enlargement, and risk of rupture after EVAR [6,30,31,32,33]. For current devices, IFU defines eligible neck diameters as ranging from 16 mm to 32 mm. All endograft designs include sizes that accommodate that wide range of proximal diameters, taking into consideration that endograft oversizing by approximately 10–20% is recommended for most designs (the Alto abdominal stent has different sizing parameters because of its unique proximal sealing feature). This oversizing is necessary to maintain the proximal seal, particularly since aortic neck dilatation after EVAR has been shown to occur in approximately 25% of all EVARs [30] and is significantly more prevalent in wide aortic necks [32]. The degree of optimal endograft oversizing has been debated. Some investigators have reported no causal relationship between excessive oversizing and postoperative neck dilatation [6], but others, including our group, have demonstrated that the degree of endograft oversizing is an independent risk factor for neck dilatation [33]. Nevertheless, neck dilatation represents disease progression rather than excessive radial force [6]. Exceeding the recommended oversizing criteria does increase the risk of device migration and subsequent development of an endoleak [34]. The dynamics between the graft and aortic wall are complex at the proximal seal, and ensuring adequate, but not excessive, oversizing is critical for a successful outcome.

### 4.4. Mural Thrombus

Mural thrombus is a common finding amongst patients with AAA, typically present in a circumferential fashion and expressed as a percentage of the neck circumference. There are no definitive criteria for what constitutes a “significant” burden, but generally >50% is considered to be clinically relevant. Data pertaining to the effect of mural thrombus on postoperative EVAR outcomes are limited and unclear. Inherently, there are apprehensions about establishing a proximal seal zone in a neck with a significant thrombus burden, especially in grafts that utilize only passive fixation. Retrospective reviews have not shown an association between neck thrombus burden and graft migration in devices utilizing suprarenal fixation [35]. Suprarenal and infrarenal thrombus do, however, introduce the risk of renal thromboembolic complications such as microembolization and sustained renal dysfunction [36,37]. It has been suggested that suprarenal thrombus and suprarenal fixation carry an especially high risk for renal dysfunction, and therefore must be considered with diligence during preoperative planning [37]. Even so, current devices have demonstrated excellent outcomes with significant infrarenal neck thrombus (presuming proper placement and adequate sealing), and this factor alone should not be prohibitive to EVAR (Table 3).

### 4.5. Conical Neck

A conical neck, defined as a neck with ≥10% diameter increase within 15 mm below the lowest renal artery, is a common morphological characteristic encountered in patients with AAA. Other morphological variants, for example, “reverse” conical neck or posterior bulge (colloquially referred to as “double bubble” configuration), pose a challenge in creating a reliable seal with proximal fixation [38,39]. Even though these morphologies fall outside of the IFU for most commercially available devices, a significant proportion of patients with these anatomies are being treated with EVAR [14,40]. There is an increased incidence of type Ia endoleaks with conical neck shapes in the setting of a short infrarenal neck [41,42]. There is a paucity of data specifically addressing conical morphology as an independent predictor of poor outcomes. One recently published retrospective analysis found no difference between rates of type Ia endoleak and graft migration in conical proximal necks versus non-hostile aortic necks. A subgroup analysis on the conical neck cohort suggested that a more aggressive oversizing strategy provided a protective benefit on graft migration, albeit not statistically significant [39]. The degree of oversizing is not clearly defined in these cases, particularly as the definition of proximal neck diameter in these anatomies varies. Surgeons should use clinical judgement to select the appropriate graft size.

### 4.6. Algorithm for Management

**Figure 1:**
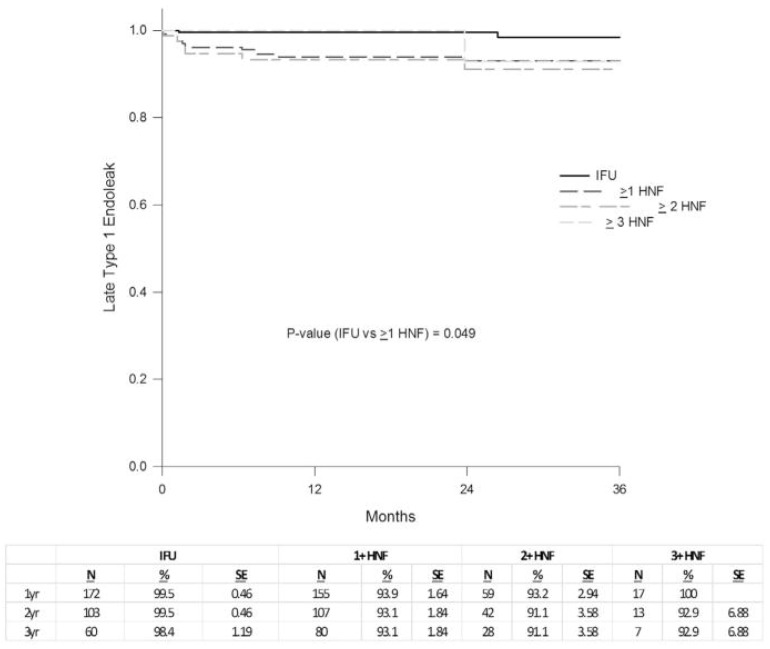
Graph from Aburahma et al. demonstrating decreased freedom from type Ia endoleaks at 36 months for patients with at least one feature of a hostile neck [43].

**Figure 2:**
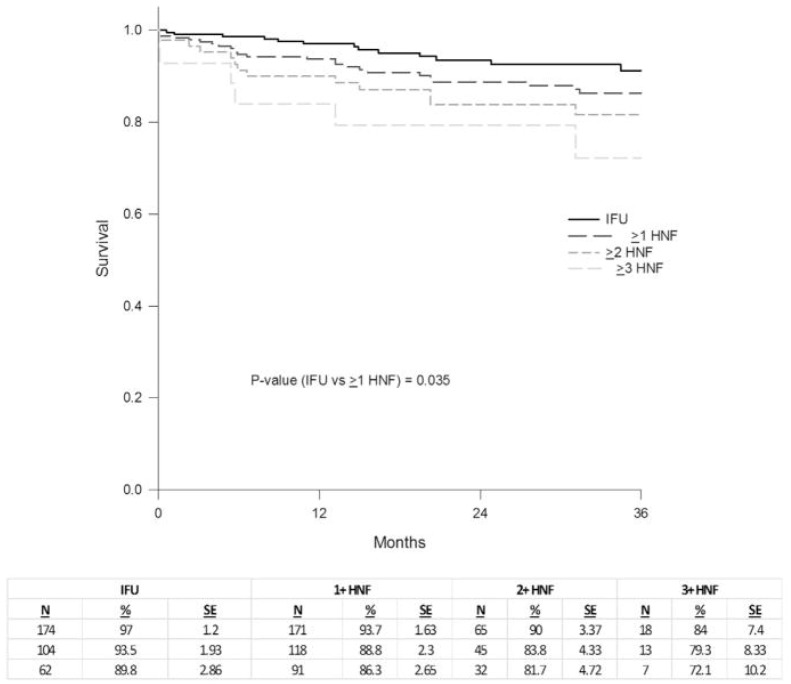
Graph from Aburahma et al. demonstrating decreased survival for patients undergoing EVAR with >1 hostile neck features [43].

**Figure 3:**
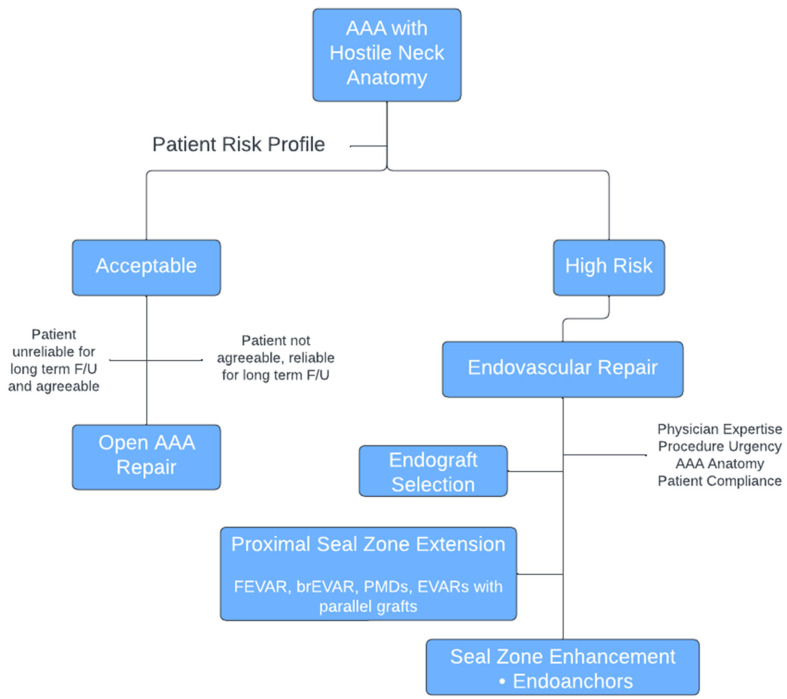
Algorithm for the management of AAAs with hostile neck anatomy.

Patients with hostile neck anatomy will likely exhibit several, if not all, of these attributes to varying degrees. Although the adverse effect of each individual hostile characteristic has been documented, the combined effect of two or more hostile features is clearly more threatening to the short- and long-term integrity of the proximal seal zone [3]. If endovascular repair is the preferred option for these patients, a thorough evaluation of the hostile characteristics and a holistic approach are paramount to preoperative planning and device selection. AAAs with hostile neck anatomy are more likely to require intraoperative adjuncts [4] as the rate of intraoperative type Ia endoleaks is higher. Furthermore, it has been shown that type Ia endoleaks occur at a higher rate not only intraoperatively, but also after long-term follow-up (Figure 1). A correlation has also been demonstrated between overall survival and EVAR in patients with hostile neck anatomy (Figure 2) [43]. The surgeon should be familiar with and be ready to use all available tools that can best overcome the limitations and risks of each instance of individual hostile neck anatomy and have a detailed discussion of all options with the patient before designing the most suitable endovascular approach for achieving the most durable outcome. Besides the individual anatomic characteristics, procedure urgency as well as the surgeon’s experience and familiarity with specific endografts and/or adjuncts and techniques weigh on the final decision. A proposed algorithm for planning the repair of an AAA with hostile neck anatomy is outlined in Figure 3.

### 4.7. Fenestrated Endografts

Custom-made fenestrated endografts and parallel grafts have been utilized to extend the proximal seal zone above the renal arteries. Fenestrated stent grafts are customized to patient anatomy and can be designed with premade branch sites. The need for the traditional landing zone of 15 mm has been obviated with these devices. Furthermore, fenestrated endografts are utilized not only for aneurysms with short necks but also for the treatment of juxtarenal and suprarenal aneurysms [44]. The Cook Zenith ZFEN device was approved for commercial use in 2012 and is currently the only fenestrated device being used in the United States. In the hands of experienced surgeons, FEVAR has exhibited promising short- and midterm outcomes. In 2021, Oderich et al. reported the results of a prospective, nonrandomized trial evaluating the 5-year outcomes of the Zenith Fenestrated stent graft for the treatment of juxtarenal AAAs in 67 patients [45]. None of these patients experienced aneurysm rupture or conversion to open repair. Freedom from all-cause mortality was 96.8%, and freedom from secondary intervention was 63.5%. Also, the incidences of type Ia endoleaks, type Ib endoleaks, device migration, and sac enlargement were low (1, 1, 2, and 4 occurrences, respectively) [45]. Katsargyris et al. demonstrated favorable outcomes of FEVAR for the repair of short neck, juxtarenal, and suprarenal aneurysms in 349 patients [46]. At five years, freedom from aneurysm-related death, target vessel patency, and freedom from reintervention were 98.8%, 98.7%, and 86.5%, respectively. Despite a considerable number of patients requiring reintervention, it is important to note that no difference in midterm mortality was observed in this group [46]. This finding is consistent with other reports that have shown no relation between secondary procedures and survival during follow-up [47]. Today, FEVAR is considered an acceptable alternative to open repair for juxtarenal AAAs in high-volume centers with experience in complex aortic repair. Their long-term durability, however, remains to be established. Similarly, the outcomes of the procedure in the hands of less experienced surgeons have not been established.

### 4.8. EndoAnchors

The Heli-FX EndoAnchor^™^ (Medtronic, Minneapolis, MN, USA) was designed to enhance endograft fixation and sealing and its use has also been shown to offer some protection against proximal neck dilatation [33]. Many of the currently commercially available endografts are compatible for endoanchor use (Table 2) [48]. The system utilizes helical implants (4.5 mm in length, 3 mm in diameter) and an applier to engage the full thickness of aortic tissue and attach the endograft to the aortic wall. Conceptually, their use is intended to replicate a hand-sewn anastomosis between a graft and native vessel; thus, they require incorporation of the adventitia for adequate strength. Endoanchors can be used primarily either prophylactically or to manage an intraoperative type Ia endoleak during the initial endograft implantation procedure. Their secondary use is when they are employed in the management of a remote type Ia endoleak. The use of endoanchors is not recommended if the sealing zone is burdened with extensive plaque, calcium, or thrombus (>2 mm thickness or >50% (180°) of the vessel circumference continuous coverage of sealing zone circumference). Also, a neck length <4 mm is unfavorable because there is limited room for safe and adequate deployment. The Food and Drug Administration cleared the product for clinical use in 2011; since approval, its use has become widespread and today the Heli-FX EndoAnchor^™^ system is still the only device on the market that utilizes endostapling technology.

The Aneurysm Treatment Using the Heli-FX Aortic Securement System Global Registry (ANCHOR) study has provided the best evidence for its use, which is suggestive of its great technical success in both index procedures and repeat interventions with proximal neck degeneration. The initial report published by Jordan et al. showed technical success in 303/319 patients (95%) and procedural success in 279 (87.5%), with an average of about six endoanchors used per patient. Furthermore, during mean follow-up of 9.3 months, 301 patients (94.4%) were free from secondary procedures with no open surgical conversions, no aneurysm-related deaths, and no aneurysm ruptures [49]. A meta-analysis by Qamhawi et al. analyzed seven EVAR and three TEVAR studies, looking at 455 and 107 EVAR patients who underwent primary and secondary fixation, respectively, as well as 66 TEVAR patients who underwent fixation [50]. Technical success in the EVAR proximal fixation group was 98.4%, in the EVAR secondary fixation group was 91.8%, and in the TEVAR group was 90.3%, with an all-cause 30-day mortality of 0.82% in the EVAR group. A safety report conducted on the safety and efficacy of Heli-FX EndoAnchors showed that it is a safe device with low rate of adverse events [51]. Based on results from the ANCHOR registry, the Endurant II and IIs (Medtronic) endografts were approved for use in 4–10 mm necks with the use of endoanchors.

### 4.9. Our EndoAnchor Experience

We recently presented a midterm review of 37 patients with hostile neck anatomy who underwent EVAR with the primary use of endoanchors in our institution. Our results indicate a durable effect on proximal seal zone integrity during a mean follow-up of 46 months, with only one remote type Ia endoleak and no ruptures in a patient cohort with an average of two hostile neck characteristics [52]. Data on outcomes related to endoanchor use compared to other complex EVAR approaches (FEVAR, parallel grafts, etc.) are sparse and future research should focus on comparing mid- and long-term outcomes in these groups.

**Figure 4:**
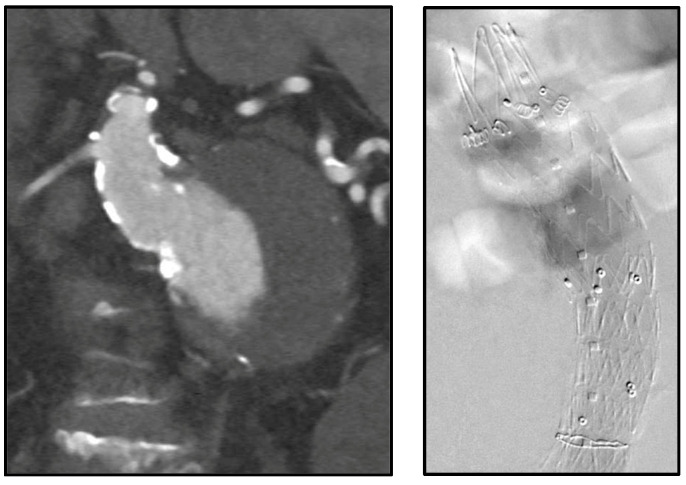
Juxtarenal abdominal aortic aneurysm with significant angulation treated with prophylactic endoanchors during index procedure.

**Figure 5:**
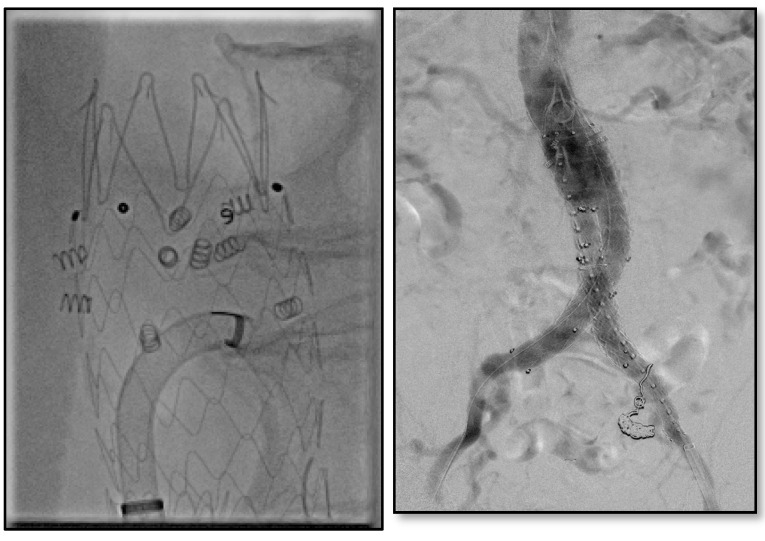
Intraoperative images of previous endograft with hostile neck anatomy (proximal neck had dilated to the same size as existing endograft) treated with eight endoanchors.

Physicians in our institution have utilized endoanchors prophylactically for hostile necks (Figure 4) and in those necessitating revisions for proximal neck dilation (Figure 5) or concern for type Ia endoleaks.

## 5. Conclusions

Hostile neck anatomy is of the utmost importance when planning an endovascular intervention for AAA. Anatomic characteristics, urgency of repair, surgeon expertise and patient preference are key determinants for optimal outcomes. Open surgical repair should still be considered for the appropriate patient.

## Figures and Tables

**Table 1:** Most widely accepted hostile neck characteristics (Delphi Consensus Group).

Neck Characteristic	Definition	Hostile Zone
Length	Distance between the lowest renal artery and the aneurysm sac	<10 mm
Angulation	Angle between the longitudinal axis of the proximal neck and the longitudinal axis of the aneurysm sac	>60°
Width	Diameter at seal zone	>28 mm
Conical shape	Diameter increase >10% compared to the immediate infrarenal diameter over the first 10 mm below the lowest main renal artery	N/A
Calcification	Calcium presence in the proximal seal zone circumference	>50%

**Table 2:** Currently available infrarenal EVAR devices in the United States in 2023.

Device	Manufacturer	FDA Approval Date	Treatable Aortic Neck Diameter	Minimum Aortic Length (mm)	Maximum Treatable Aortic Neck Angle	Proximal Fixation	Native Iliac Diameter (mm)	Unique Features
Excluder	Gore	2002	19–32	15	<60	Infrarenal	10–18.5	Repositionable delivery system allows ability to recapture and reposition body
Zenith Flex	Cook	2003	18–32	15	<60	Suprarenal	8–20	Spiral Z flexible limbs
Endurant II/IIs	Medtronic	2010	19–32	10	<60	Suprarenal	8–25	Allows for short (10 mm) neck
AFX2	Endologix	2011	18–32	15	<60	Infrarenal or Suprarenal	10–23	Unibody component allows for anatomic fixation on aortic bifurcation
Aorfix	Lombard	2013	19–29	15	<90	Infrarenal	8–19	Ability to treat <90-degree angulation
Alto	Endologix	2020	16–30	7	<60	Suprarenal	8–25	Allows for short (7 mm) neck
Treo	Terumo	2020	17–32	15	<60	Suprarenal and Infrarenal	8–20	Minimize modular disconnection; allows for late repositioning

**Table 3:** Currently available, compatible AAA endograft systems as per instructions for use with Medtronic Heli-FX ^™^ EndoAnchors.

Device	Manufacturer
Zenith	Cook
Excluder	Gore
Endurant	Medtronic
